# Intestinal Concretions

**Published:** 1859-03

**Authors:** F. K. Bailey

**Affiliations:** Joliet, Ill.


### ARTICLE VI.

### INTESTINAL CONCRETIONS.

BY F. K. BAILEY, M.D., OF JOLIET, ILL.

Was called Saturday evening, January 9th, 1858, to see Mrs. G., aged 60. She had complained for nearly twenty-four hours of severe pain in the left hypogastric region, attended with great nausea, and constant vomiting of whatever was swallowed, immediately after being taken.

The tongue was coated at the middle and base, and red at the tip. Pulse soft, and not over ninety in a minute. There had been no chill, nor febrile excitement since the attack.

I had been called two or three times, within six or eight months previous, to prescribe for a severe pain in the region of the stomach, of a neuralgic character, which had yielded to the prompt use of anodynes. She had complained of this pain in the stomach, at times, about a year previous to her removal to the city. She was obliged to exercise great caution in the use of many articles of diet, which by most people would be borne without inconvenience. To allay this pain, she had acquired the habit of taking morphine. To obviate the constipating effects of the morphine, she had taken cathartics of different kinds.

Never until the present time, however, had vomiting attended the paroxysms of pain from which she had suffered so much. A laxative, by anodynes, had sufficed to produce relief.

At this time the symptoms were unlike those of any previous attack. The pain was lower, very intense, with a great degree of tenderness upon pressure, and inability to turn in bed.

Organic disease seemed to have existed, to cause such paroxysms of pain in months past, and the severe nausea and vomiting at this time, attended with a soft and slow pulse, caused apprehensions of a fatal result. Prescribed powders composed of pulv. opii and saccharine ext. aconite, each one grain, to be given every two hours, with sinapisms to the seat of pain and the lower extremities, and mucilaginous drinks.

*Sunday morning*, 10 *o'clock*.—No better; vomiting has continued through the night, with no abatement of the local distress. No evacuation from the bowels or bladder. The ejection from the stomach consisted of drinks, and a dark green substance floating upon the surface. Pulse same as last evening. To continue same powders, with the addition of a grain of calomel, every four hours. To administer enemata of solution of common salt, with the addition of ʒij. oil turpentine.

*Monday morning*, 11 *o'clock*.—No better. Still rejects drinks, with the green secretion. Nothing retained upon the stomach, and no evacuation from bowels or bladder. Tongue more coated and thirst more intense. Begs for drink, and swallows only to reject it from the stomach. Pulse eighty, and occasionally intermitting. Becoming weaker every hour. To continue powders, and applied a belladonna plaster to the seat of pain. Seven o'clock, evening, no change. Enemata passed off with no effect. Pulse seventy-five, feeble and intermitting. Discontinue powders, and to give nothing but flax-seed water through the night, with a little gruel.

*Tuesday morning.*—Pain still continues, with occasional distress in the region of the liver. Prescribed powders of ext. aconite 1 grain, sul. morphine $\frac{1}{6}$ grain, calomel 5 grains, once in four hours, mixed with pulverized sugar, and placed dry upon the tongue. *Evening.*—Feels somewhat better. She thought the first powder produced relief. Has vomited, however, through the day, and during the evening ejected a quantity of yellowish green substance, and, for the first time since the attack, drops of sweat were seen upon the face.

A small quantity of dark urine was voided during the afternoon, the first for some days. To discontinue the powders, of which she had taken three, and give injections of salts and senna infusion during the night. To take mucilaginous drinks and gruel.

*Wednesday morning.*—Nausea not so great, but has vomited at times through the night, and the substance ejected has a decided fæcal smell. Slight evacuation from the bowels this morning, of a bilious character, and very offensive. To take nothing through the day but water and gruel, and to be kept as quiet as possible.

*Evening.*—Has not vomited much through the day. Pain less severe. Slight evacuation from bowels, and urine more copious. Has been very thirsty.

The pain is "working down," to use the patient's words. Pulse fuller and stronger. Can take rice water with some relish. Turns in bed with less difficulty, and is evidently improving.

*Thursday morning.*—Has slept some during the past night. Free evacuations from the bowels. Nausea nearly ceased, and pain much lessened. To have some beef broth.

*Friday morning.*—Much improved, and begins to relish food; has passed a quantity of hardened fæces; and feels free from nausea.

*Saturday morning.*—Still better. Has slept very well during the night. Last evening she voided from the rectum, a solid, oval-shaped mass, weighing 183 grains, and about the color of rhubarb root. The specimen is herewith presented to the members of the society for inspection.

From this time the patient continued to improve, and up to the date of this paper, has enjoyed better health than she had for some years.

We have, in the case before us, one presenting the usual symptoms of intestinal obstruction, with the evident cause of the obstruction voided. The severity of the symptoms, and fortunate recovery, with the voiding of the concretion above mentioned, rendered the case one of much interest at the time of its occurrence. The works upon practical medicine speak of intestinal concretions as constituting a cause of obstruction. The above case presented many of the usual appearances seen in that morbid condition.

The great depression of vital energy, constant vomiting, and other symptoms, threatened immediate death, and such would have been the result, had not the mass been dislodged and expelled. From the previous history of the case, it is probable that it had been a source of trouble for nearly two years, and perhaps for a greater length of time.

It was not our design that this article should be published in the transactions, when read to the society, but merely to accompany the exhibition of the specimen.

